# Urban Stone Marten (*Martes foina*)–Human Conflicts: A Longitudinal Survey‐Based Approach to a Persistent Challenge

**DOI:** 10.1002/ece3.74294

**Published:** 2026-09-04

**Authors:** Michał Strączyński, Sayantani M. Basak, Paweł Kwapisz, Joanna Tusznio, Jesse S. Lewis, Izabela A. Wierzbowska

**Affiliations:** ^1^ Institute of Environmental Sciences, Faculty of Biology Jagiellonian University Krakow Poland; ^2^ Doctoral School of Exact and Natural Sciences Jagiellonian University Krakow Poland; ^3^ School of Biological, Earth and Environmental Sciences University College Cork Cork Ireland; ^4^ Krakow Municipal Greenery Authority, Forest and Nature Team Krakow Poland; ^5^ College of Integrative Sciences and Arts Arizona State University Mesa Arizona USA

**Keywords:** car mechanics, human dimension, interview, public perception, urban ecology

## Abstract

Although stone martens ( *Martes foina* ) are widely recognised for causing urban property damage, including damage done when entering and tearing up car engine compartments, the human dimensions of these conflicts remain largely understudied. Based on interviews with local car mechanics in Krakow, Poland conducted in 2010 and 2023, we explored the long‐term human‐stone marten interaction through the lens of the car damage conflict. Contrary to expectations of increased conflicts with rising car registrations, the research found that while the total number of reported incidents slightly increased from 1135 in 2010 to 1219 in 2023, it did not differ significantly (*p* = 0.8) between the years. The conflict rate, when adjusted for the number of registered cars, significantly decreased. Specifically, the adjusted conflict count dropped from 321 in 2010 to 270 in 2023 (*p* < 0.01), despite continued urban expansion and vehicle ownership growth. A notable shift in 2023 was the official inclusion of marten repellents by car manufacturers, indicating the perceived importance of this issue. Car owners, even more than interviewed mechanics, appeared to view stone martens as a growing threat, leading to increased use of countermeasures and an increase in their availability at licensed providers, although their effectiveness lacked consensus among respondents. The study concludes by advocating for future research to prioritise stone martens, recognising their overlooked importance in urban human‐wildlife conflict studies.

## Graphical abstract

Interviews with car mechanics in Kraków (2010 vs. 2023) examined long‐term human–stone marten conflict related to car engine damage. While the total number of reported incidents changed little, the conflict rate relative to the growing number of registered cars significantly decreased. Despite this decline, perceptions of martens as a threat have increased, reflected in greater use and availability of repellents and their recent inclusion by car manufacturers.

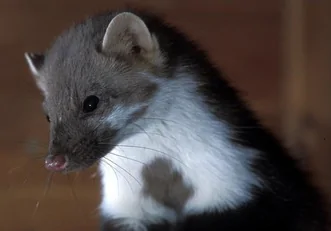

## 1 Introduction

In the current day, following a rapid expansion of cities, nearly 55% of the human population lives in urbanised areas as of 2019, with a potential to increase up to 68% by the year 2050 (United Nations 2019). The rapid growth of urbanisation leads not only to habitat fragmentation and biodiversity loss (Theodorou 2022), but also creates distinct, urban ecosystems characterised by a variable degree of human modification and high intensity of human‐wildlife interactions (Soulsbury and White 2015). Human interactions with wildlife are increasing globally due to the habitats and daily activity of people and wildlife overlapping in space and time, especially as animals recolonise urbanised areas (Magle et al. 2019).

The study of human‐wildlife interactions increasingly incorporates social psychology to bridge ecological knowledge and human behaviour, forming the core of human dimensions of wildlife (HDW) research. Central to this field is the cognitive hierarchy framework, which ranges from abstract values to specific behavioural intentions. Two primary wildlife value orientations have been identified: a domination orientation, viewing wildlife as a resource to be managed for human benefit, and a mutualism orientation, viewing wildlife as deserving of rights and care (Manfredo 2008; Teel and Manfredo 2010). These orientations shape attitudes and predict human behaviour. Emotional responses, driven by both learned and innate psychological mechanisms, interact with cognitive states to produce the full range of human reactions to wildlife—whether toward an abstract concept or during direct encounter, studying which is a large part of HDW research (Jacobs et al. 2012).

Human‐wildlife interactions are varied (Bhatia et al. 2020) and can be positive when they produce positive emotional responses, such as when they involve human appreciation of wildlife or when people value the existence of the wildlife in their local area so much that they are willing to accept the potential and actual costs of living with wildlife, also called tolerance toward wildlife. Tolerance in human‐wildlife conflict research lacks a consistent definition (Brenner and Metcalf 2020). Some define it as a positive attitude toward a species despite the damage it causes (Lewis et al. 2012), others as a neutral midpoint between active hostility and stewardship (Bruskotter and Fulton 2012), and others still as a more nuanced, fluid attitude shaped by beliefs, emotions, and behavioural dispositions working together (Frank 2016). Within HDW research, wildlife acceptance capacity (WAC) is commonly used as a proxy for tolerance, typically measured by asking whether wildlife populations should grow, shrink, or remain stable (Bruskotter et al. 2015). A complementary approach assesses attitudes toward specific management actions in response to conflict, revealing thresholds of acceptability for particular wildlife behaviours (Morzillo and Needham 2015). A streamlined, working definition of tolerance drawing from HDW, sociology, and animal behaviour knowledge was proposed by Brenner and Metcalf (2020) as “accepting wildlife and/or wildlife behaviours that one dislikes.” Kansky et al. (2016) found that exposure and direct experiences of wildlife—both positive and negative—can further increase or decrease the threshold of acceptable costs. Studying the human experience and exposure to wildlife is thus important for establishing existing tolerance levels and implementing methods of increasing it.

However, negative interactions between people—individuals or groups—and wildlife are also increasing globally, which are classified as human‐wildlife conflicts (hereafter: HWC) (König et al. 2021). A HWC occurs when material and non‐material costs related to wildlife exceed the human tolerance threshold (Dickman 2010; Dickman et al. 2011; Soulsbury and White 2015). Common HWC include competition for resources, direct assault on humans, but also capture, vehicle collisions and property damage, especially in urbanised areas (Nyhus 2016), which are an emergent location for these conflicts (Soulsbury and White 2015; Conover 2019). HWC can also result from indirect drivers, without a direct interaction between people and wildlife, or material damage. In such cases, HWC are embedded in human beliefs and emotions, stemming from subjective assessments of potential risk based on the knowledge of presence or sightings of wildlife (Basak et al. 2022). Risk perception is an important driver of HWC, inherently subjective and translating to a variety of human reactions (Slovic 2015).

Species most commonly associated with HWC in Europe include ungulates such as red deer ( *Cervus elaphus* ), roe deer ( *Capreolus capreolus* ), elk ( *Alces alces* ), and wild boar ( *Sus scrofa* ) (Saint‐Andrieux et al. 2018; Laliberté and St‐Laurent 2020) and carnivores such as red fox ( *Vulpes vulpes* ), stone marten ( *Martes foina* ) (Basak et al. 2020; Wierzbowska and Lesiak 2020), and pine marten ( *Martes martes* ) (Birks and Brown 2006). As urban ecosystems grow in size, HWCs are increasingly experienced not only in traditionally recognised contexts such as human use of wild and agricultural areas, but also entirely new, distinct contexts only present in urban environments. Urban HWCs are characterised by a large degree of spatio‐temporal heterogeneity dictated by the quickly changing anthropogenic environments. This causes a variety of types of conflicts with different species to be confined in a relatively limited space of a city, often requiring vastly different management strategies (Basak et al. 2020). Urban HWCs with individual species have also been shown to change in intensity, with certain species becoming more conflictual over the years, while others became marginal in public perceptions (Basak et al. 2022), necessitating a future‐proof approach to their management.

The probability of conflicts could also be influenced by how wildlife are influenced by humans and development, as wildlife species can be broadly grouped into three categories based on their response to urbanisation (Blair 2001). Species highly sensitive to human disturbance and habitat change, such as some large herbivores (e.g., such as deer and bison) and carnivores (e.g., cougar [ *Puma concolor* ], Eurasian lynx [ *Lynx lynx* ] brown bear [ *Ursus arctos* ] and grey wolf [ *Canis lupus* ]) are characterised as “avoiders” and are often avoid using highly developed areas (Mckinney 2002; Kaim et al. 2025). Species that use suburban or moderately urbanised environments, such as red foxes ( *Vulpes vulpes* ) and other mesocarnivores are categorised as “adapters”. Lastly, species that use highly developed environments are categorised as “exploiters” (e.g., the rock dove [ *Columba livia* ] and brown rat [ *Rattus norvegicus* ]; Mckinney 2002). Mustelids were shown to enter and forage in a variety of urbanised areas, making them important adapters which utilise the resources available there, but do not depending on them exclusively for survival (Červinka et al. 2014). Mustelid species inhabiting urban environments are also an important study subject in terms of the possible HWC following their colonisation of cities, especially health risks associated with the transmission of parasites (Łopucki et al. 2019). They remain, however, understudied in the context of their long‐term dynamics, impact on human well‐being and effectiveness of mitigation measures (Magle et al. 2019).

The stone marten is one of the most widespread mustelids occurring in urbanised areas throughout Europe (Maran et al. 2015) and also associated with HWCs in many cities. Stone martens were first observed colonising European cities in the 1950s (Krauze‐Gryz et al. 2024). Their role in urban environments is that of an opportunistic predator, with a flexible diet consisting of fruit, birds and small mammals with relatively low reliance on direct feeding by humans and scavenging from anthropogenic refuse (Herr 2010). The diet and hunting behaviour of stone martens directly affect the urban environments by influencing other animals, such as by affecting birds nesting in cities and urban rodent populations through direct predation and top‐down regulation of foraging and breeding behaviour (Preisser et al. 2005). In this way, urban stone martens may play a role in regulating urban rodent populations, which are themselves a more direct source of conflict with humans. The stone marten's behavioural flexibility allows it to adapt to urban environments by establishing populations of higher densities with smaller but overlapping territories which is rare for the conspecifics in non‐urbanised areas (Herr et al. 2009b).

Stone martens can occur in more highly developed areas when compared to other species, such as the red fox, and are well adapted to urban living due to their effective use of urban resources, especially anthropogenic shelters (Krauze‐Gryz et al. 2024). An individual stone marten utilising anthropogenic refuges often shelters in multiple dens, many of which could be placed in inhabited buildings, especially during winter (Herr et al. 2010). Stone martens enter car engine compartments, where they seek cover, reside for short periods of time, and may chew through the wiring and other engine parts of vehicles. In Germany, this generated economic losses of close to 60 million euros in 2013 and 128 million euros in 2023 (German Insurance Association 2023). This type of damage from stone martens was reported a few decades ago (Herr 2010) and has led to the introduction of diverse repellents aimed at reducing the risks to vehicle engines. Although the stone marten is frequently cited as a common conflict species (Basak et al. 2020) and its presence in urban environments often leads to various forms of damage (Wierzbowska et al. 2017), their surrounding circumstances are still insufficiently documented.

In Poland, hunting stone martens near human habitation or otherwise killing them is illegal without permission (Minister Środowiska 2016). Urban citizens are therefore limited in their response to the conflicts with stone martens and can only use non‐lethal repellents as a countermeasure. In Europe, the commonly used repellents can be divided into two main categories: electric instruments involving flashing lights, noise generators which operate by emitting high‐pitched sounds that irritate the animal, making it less likely to enter the engine compartment as well as chemical‐based repellents which usually involve smelly sprays that are unpleasant to the marten or come from predators, such as the domestic dog ( *Canis lupus familiaris* ) and scare it away by informing about potential danger (Birks 2017). Another method was to include the installation of steel meshes and cardboard panels that prevent physical access to the car engine compartment (Broekhuizen et al. 2010; Birks 2017).

Although the methods used for conflict mitigation are diverse, there is a general lack of research into urban mustelids (Streicher et al. 2023), and the material damage caused by them as well as possible mitigation methods are especially weakly investigated. The damage caused by stone martens to car engines is widespread, frequently highlighted in the media (Dziatkiewicz 2009; Szypulski 2024) and everyday conversations. Despite the scientific community's awareness of this problem since the 1970s (Herr et al. 2009a), and the species' broad distribution throughout Europe, contemporary research that specifically investigates the relationship between stone martens and cars is remarkably scarce (Herr et al. 2009a; Tóth et al. 2009). Surprisingly, some studies addressing conflicts with stone martens fail to mention this issue altogether, as highlighted by Peeva and Raichev (2016). Recent survey studies conducted in 2020 among wildlife professionals and the residents of the largest German cities by Moesch et al. (2024) emphasise the critical role of this interaction as a primary conflict with the species, underscoring their status as a significant nuisance species. This highlights a gap in our understanding, particularly concerning the human dimensions of this conflict and potential mitigation strategies. Recent study (Basak et al. 2026) focused on prioritising future research on human‐wildlife conflicts highlights urban stone martens specifically as understudied, showcasing the need to further assess their status regarding risks, nuisances, and conflicts. Mitigating conflicts through changing human relationship and behaviour toward wildlife relies upon successful messaging, emphasising the role of individual action in limiting human–wildlife conflicts, building self‐reliance and confidence in the actions taken (Puri et al. 2024). Providing people with clear, informative signs and instructions aimed at modifying their behaviour was also shown to be an important step in reducing conflicts (Griffin et al. 2022).

The human dimensions approach to HWCs focuses on gathering accurate and comprehensive data that explains human thoughts and behaviours concerning wildlife, utilising the principles and methodologies used in social sciences. Further, it involves determining how to effectively utilise this information in the decision‐making process regarding wildlife in the future (Manfredo and Vaske 1995).

Researching human dimensions of specific HWCs requires understanding perceptions, expectations and experiences of stakeholders directly affected by them (Nyhus 2016). Stakeholders are defined as individuals and institutions impacted by wildlife, involved in its protection, management or those simply interested in wildlife. Even stakeholders nominally belonging to the same interest group (such as “farmers” or “conservationists”) can hold a wide range of unique and opposing views on any given matter connected to wildlife, and thus exploration of human‐wildlife coexistence should seek out and highlight such nuances (Kiffner et al. 2025). In the HWC with stone martens, the main stakeholders are car owners, as well as those working with the damaged property, such as car mechanics, or those offering solutions, such as car manufacturing companies and repellent producers. To our best knowledge, none of the previously stated stakeholders' perceptions on this issue have been studied before. For the first ever exploratory study on this topic, car mechanics appear as the most important group due to possessing unique perspectives and experiences which can help identify multiple aspects of this conflict, such as types of damage, its costs, changes through time, the factors underpinning it and ways of mitigation. For that reason, they can be identified as local knowledge holders (Ulicsni et al. 2019; Wheeler and Root‐Bernstein 2020), whose work requires combining experiences from many individual cases of damage and linking other interested parties, such as car owners, car producers and producers of repellents.

Therefore, the main aim of this study was to assess how stone marten–related car damage conflicts have changed over time in Poland's second‐largest city (Krakow), using comparative in‐depth interviews with car mechanics conducted in 2010 and again in 2023. This aim was achieved by the following four specific objectives:

1. Firstly, to identify the car mechanic's perception of the scale of the conflict with stone martens.
2. Secondly, to understand their views on factors potentially causing or influencing the occurrence of car damage.
3. Thirdly, to recognise the perceived severity of the conflict through the types of car damage and their cost estimates.
4. Finally, to ascertain the stakeholders' perspectives on conflict mitigation based on the reported availability, use and perceived effectiveness of the mitigation methods and tools used.

This study will thus help address existing knowledge gaps regarding the human dimensions of stone marten conflict, offering insights essential for developing effective mitigation strategies and fostering future tolerance of the species.

## 2 Methods

### 2.1 Target Species and Study Area

**FIGURE 1:**
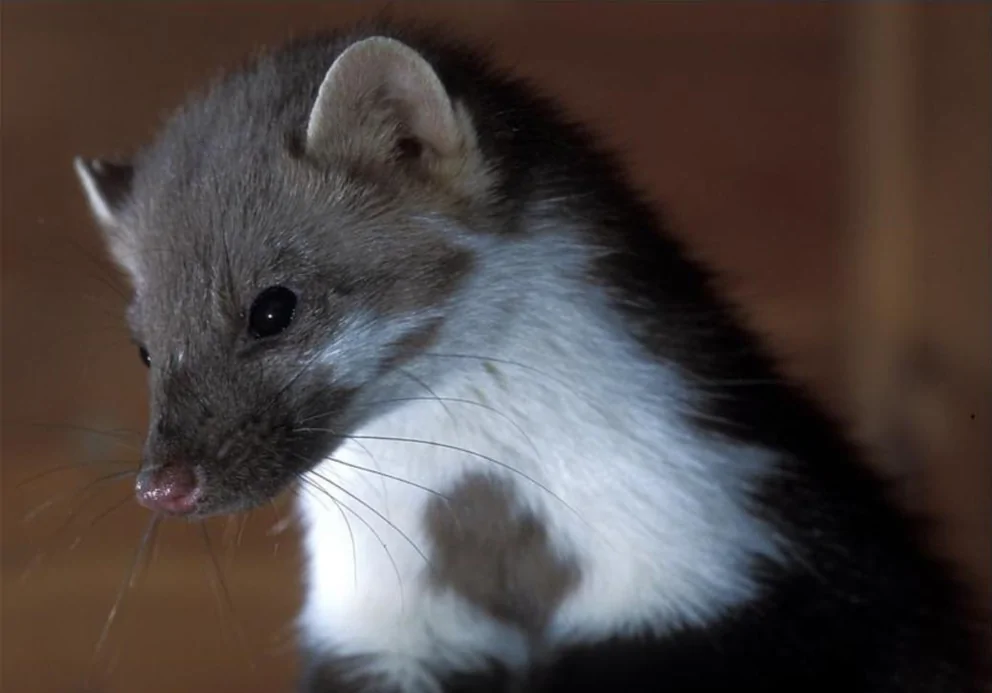
An image of the stone marten ( *Martes foina* ). *Source:* IUCN (Maran et al. 2015).

The population numbers of the target species, that is, the stone marten (Figure 1), in Krakow did not change over the years and were estimated at 679 in the year 2016 and 611 in the year 2023 (Office of Forest Management and Forest Geodesy 2016). Furthermore, between the years 2010 and 2023, the number of cars registered in the city increased from 353,540 in 2010 to 588,856 in 2023 (Kasperkiewicz 2023).

**FIGURE 2:**
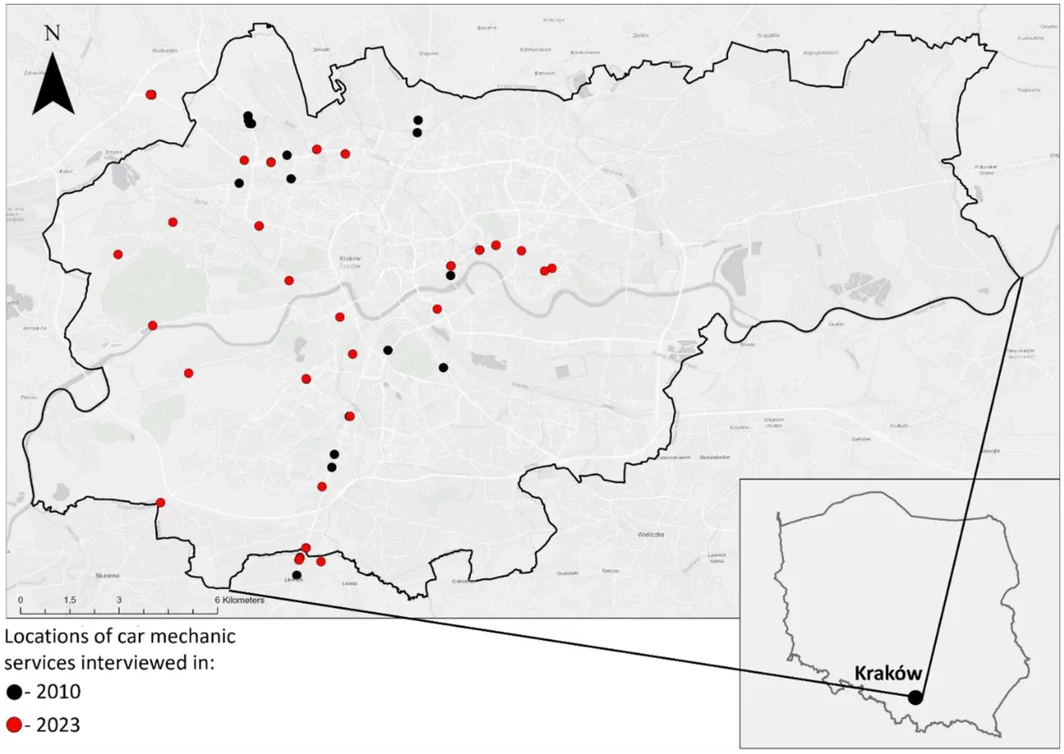
The localisations of surveyed car mechanic services in 2010 and 2023 in the study area of Kraków, Poland (50°03′42″ N 19°56′15″ E).

Krakow, where the study was conducted (50°02′59′′ N and 19°56′40′′ E), is the second‐largest city in Poland, encompassing an area of 327 km^2^ in southern Poland (Figure 2). It had a human population of 756,200 in 2010 (Chełstowska and Kulczycki 2011) and nearly 804,000 by 2023 (Juda and Rudnik 2023). Land cover is varied, comprising urbanised terrain, green areas, and agricultural lands. In the year 2010, 33.6% of the city area was urbanised, 45.7% was agricultural, with the remaining territory (20.7%) being distributed between other types of ground cover such as forests and water bodies (Chełstowska 2011). Over the years, there was a notable increase in urbanisation: in 2023, 48.2% of the city is built‐up urbanised land, while 42.4% of the city grounds are considered agricultural. The remaining 10% of Krakow is made up of green areas such as forests, parks, nature reserves and Natura 2000 areas as well as water bodies (Kozień and Sochacka 2023). The city is set on the banks of the Vistula River, which functions as a potential migration corridor for multiple species (Romanowski 2007).

### 2.2 Data Collection

#### 2.2.1 Interview Design

We selected key informant interviewing as our qualitative data collection technique because it enables researchers to more accurately document the experiences and perceptions of decision‐makers, community members, and those knowledgeable on a given topic (Lucas et al. 2022). The data was collected through interviews with workers at car mechanic service centres (hereafter car mechanics), involving both closed and open‐ended questions (Appendix A). They opened the possibility of asking follow‐up questions (Hyman and Sierra 2016). We conducted the interviews around the four research objectives: the analysis of views on the scale of the conflict with stone martens, factors potentially causing or influencing the conflict, the cost of the damages done by martens, and the availability and use of mitigation methods.

#### 2.2.2 Selection of the Participants

We selected 30 participants from diverse car mechanic service centres in the city. In 2010, the participants were selected from among authorised car mechanic services for major manufacturer brands across the city (Figure 2). One worker was chosen from each car mechanic service centre, and the authors made sure the interview was conducted with the knowledge and consent of the car mechanic service owner or a currently available manager. The chosen interview methodology was implemented as outlined in the Jagiellonian University's Instructions for applicants regarding research that does not require an ethical overview (Jagiellonian University 2023) and did not require additional ethical consultation or permissions. In 2023, we attempted to interview from the same 30 car mechanic centres; however, we were only partially successful (*n* = 13 repetitions out of 30), as many were either closed down permanently or otherwise unavailable using the contact information provided on their websites. Therefore, we chose 17 new car mechanic service centres which were located closest to the unavailable ones.

#### 2.2.3 Interview Process

The interviews were first conducted face‐to‐face in February 2010 and repeated in March and April 2023. However, if a participant did not wish or was unable to meet in person, a telephonic interview was conducted. During each interview, specific, standardised questions were asked (Appendix A). A strong emphasis was placed on the free‐flowing conversation with the interviewee to gather as much additional detail and insight as possible. Individual interviews varied in length and lasted between 10 and 20 min. The interviews were conducted and transcribed in Polish, and later translated into English by the first author, who is a native Polish speaker. After transcription, the texts were summarised in English for further analysis. The names of car mechanic service centres have been redacted for data protection, while the general regions of the city in which they were located can be found in Appendix B, keeping the interviewees anonymous.

### 2.3 Data Analysis

To identify the statistical differences in reported numbers of the stone marten conflicts between 2010 and 2023 (objective 1), a Chi‐squared test was performed. To do so, we first normalised the data with the number of registered cars with the following Equation (1).

(1) $$x=\frac{\text{number of reported conflicts in}\;i^{\mathrm{th}}\;\text{year}}{\text{number of registered cars in}\;i^{\mathrm{th}}\;\text{year}}*100000$$

where *x* is the number of reported conflicts per 100,000 registered cars and *i* is the year 2010 or 2023.

Furthermore, the effect size was used to evaluate the magnitude of the difference between the years. Calculating the effect size allows for presenting the strength of the reported effects in a standardised metric that can be understood regardless of the sample sizes used, allowing not only to report statistical significance but also practical significance (Kearney 2017) and is used successfully in social survey studies (Basak et al. 2022). One of the most commonly used measures of effect size for Chi‐square tests is Cramér's V (McHugh 2013). Cramér's V values range from 0 to 1, where higher values indicate a greater effect size based on the degrees of freedom (Cramér 1946). For the exact interpretation of the values used, see table 2 in Kim (2017). To investigate if car mechanic services interviewed both in 2010 and 2023 showed a shift in reported numbers of damage incidents and experiences over the years, the Wilcoxon Signed Rank test was performed on the numbers they provided in both years.

To compare the number of reported conflicts between the seasons (objective 2) and the number of reported damaged car parts (objective 3) in both years, a Chi‐squared test was performed, and the effect size was calculated. In Poland, calendar seasons are categorised as spring (March–May), summer (June–August), autumn (September–November) and winter (December–February) (timeanddate.com 2026). Because some answers regarding the seasonality of the conflict with stone martens were given as the exact month when the conflict was particularly prominent, whereas others consisted of the general season or 2 months out of the seasons, all answers were coded as giving a season or seasons corresponding to the respective months. All calculations were performed in the R environment (ver.4.03) (R Core Team 2024).

To identify the reported availability and use of mitigation methods (objective 4), we used MAXQDA Analytics Pro, a qualitative data analysis software. We used open coding for developing categories of thought and other aspects relevant to our respondents—codes were developed based on the topics appearing in interviews while reading the transcriptions and assigned to relevant parts of sentences. The number of codes in the document represents the number of times a particular topic was mentioned by respondents. Open coding was used to provide a structure for the comparison of textual data from the interview transcriptions. Other studies researching the management of wildlife conflicts have used similar methodologies to code interviews (Luat‐Hūʻeu et al. 2023). The code co‐occurrence map created in the same program was used to help conceptualise the narratives expressed in the interviews and structure the narrative of qualitative analysis. The code co‐occurrence map was calculated based on the presence of codes in the same interview transcriptions. It represents the number of analysed categories and the number of co‐occurrences—the presence of a pair of codes in the same transcription. The coding of the interview transcripts and the creation of the code map were performed primarily by the first author, consulted with co‐authors at each step (code proposition, final coding with the complete list of codes) in order to assess the validity of emerging themes and readjust them to the research questions as needed. Themes were identified while the first reading of the transcriptions, when respondents' statements appeared relevant for the research questions; either a new code was proposed and assigned to the statement, or an existing, already developed code was assigned to the relevant part of the text.

## 3 Results

A total of 30 respondents were interviewed both in 2010 and 2023. All of the respondents were male. According to the respondents, their clients overwhelmingly came from their local area—usually the surrounding neighbourhood.

### 3.1 Perception of the Scale of the Conflict With Stone Martens

In 2010, out of 30 respondents, 26 reported encountering marten damage in the engine compartments of serviced cars. In 2010, there were a total of 1135 reported cases of vehicle damage by martens with an annual average of 37.83 (SD ±40.96) per car mechanic service (Table 1). After normalisation of data, the number of reported conflicts was 321 per 100,000 registered cars.

**TABLE 1:** The frequencies of conflicts reported at each car mechanic service in 2010 and 2023.

2010		2023	
ID	Reported conflicts	ID	Reported conflicts
A1	20	B1	50
A2	10	B2	24
A3	20	B3	4
A4	10	B4	12
A5	50	B5	220
A6	12	B6	270
A7	90	B7	0
A8	120	B8	0
A9	60	B9	40
A10	50	B10	30
A11	2	B11	50
A12	0	B12	156
A13	20	B13	15
A14	20	B14	120
A15	0	B15	20
A16	30	B16	48
A17	5	B17	20
A18	120	B18	0
A19	20	B19	18
A20	100	B20	20
A21	0	B21	0
A22	4	B22	0
A23	0	B23	42
A24	80	B24	24
A25	50	B25	4
A26	150	B26	12
A27	12	B27	12
A28	15	B28	0
A29	15	B29	0
A30	50	B30	8
Total	1135	Total	1219
Mean	37.83	Mean	43.53

*Note:* A = Respondents in 2010; B = Respondents in 2023.

In 2023, 25 respondents reported encountering marten damage. The total number of conflicts reported was 1219, with an annual average of 43.53 (SD ±65.99) per respondent (Table 1). After the normalisation of data, the number of reported conflicts was 270 per 100,000 registered cars. Even though there was an observed rise in the number of reported conflicts (from 1135 in 2010 to 1219 in 2023), there were no significant differences between the years (*p* = 0.8). However, when adjusted with the number of registered cars, conflicts have significantly reduced (from 321 in 2010 to 270 in 2023) (*χ* ^2^ = 24.61, df = 1, *p* < 0.01). The effect size was high (*V* = 0.64, df = 1), indicating a substantial difference.

Within the services interviewed repeatedly, the average number of reported incidents was equal to 35.69 in 2010 (SD ±35.18) and 67 in 2023 (SD ±85.82). The results of the Wilcoxon Signed‐Rank test indicated that there is a non‐significant, small difference between numbers reported by each individual service in 2010 (Mdn = 20, *n* = 13) and 2023 (Mdn = 30, *n* = 13), *Z* = 0.6, *p* = 0.529, *r* = 0.2.

When asked to describe their observations on the number of cars damaged by the martens, the respondents' personal views on the frequency of the conflict varied. In 2023, some (*n* = 6) claimed the problem is severe and might be getting worse: “The issue is big, and it keeps getting bigger, I feel like there is constantly more and more damage done, we see it in many, many vehicles”‐one said. Another commented: “The issue is very persistent, and we see an increase in damage done. It doesn't go away.”

Another view, however, expressed by a similar number (*n* = 7) of respondents, is that the problem of damage in car engine compartments is small and not very impactful. “It happens, yes. But the situation really is not bad. We find traces of animal presence in the vehicles. Damage? Only very rarely. And even if it happens, the damage is not very substantial. Usually, it is only visual: some tear or bite marks on the sound‐proofing elements or scratches here and there,” explained another respondent. Another group (*n* = 6) of vehicle mechanics claimed it is hard to say how large the problem is, as they do not have any statistics on how many vehicles are damaged by martens. This group was excluded from the statistical analysis of the scope of the conflict but was taken into consideration when forming the qualitative narratives about respondent perceptions. Interestingly, one respondent mentioned that while he does not frequently encounter marten damage in cars, his clients talk about them when discussing their vehicles: “I cannot say I have seen much. Sometimes we have a cable chewed on or some paw prints, but it's rare. However, when I talk to my clients, I hear about their past experiences with marten damage even if I don't see it myself. And this seems to be an epidemic”.

Most mechanics did not provide information on the total number of vehicles they service annually, either because they do not track these figures or because they consider them trade secrets, making it difficult to compare the proportion of cars affected by marten damage across different workshops. Still, according to some of the respondents both in 2010 and 2023, anywhere between 50% and 70% of all vehicles had some traces of marten presence such as paw prints, food remains and scats. The following words were used in describing the scale: “habitual”, “occasionally”, “large”, and “often”, but also “not bad”, “sporadic”, “little.” No changes in descriptions of the scale of conflict were found between the years during the repeated interviews of car mechanics.

### 3.2 Views on Factors Potentially Causing or Influencing the Occurrence of Car Damage

We asked various questions about the cars being brought for repair due to marten damage, such as, “Which car models were brought in because of martens the most/least often?”, “Do you know where the damaged cars were parked?” and “Do you have any additional observations regarding the damage?” In 2010, neither the model nor the age of the car was said to influence the conflict. In 2023, a few respondents (*n* = 4) mentioned the age of the car as a factor. Only one of the interviewees mentioned the car's model as a factor which might make it more likely to be damaged by martens but provided no further details.

In 2010, parking was generally pointed to as the most important factor dictating the conflict. In 2023, parking outside, in the streets, was often (*n* = 13) cited as greatly impacting the risk of a car being damaged. Some (*n* = 2) respondents claimed that even parking a car in a garage was insufficient, as martens can still find ways of accessing their engine compartment. The interview participants in 2010 did not describe additional car‐related factors. In 2023, (*n* = 5) respondents pointed to the potential role of materials used in vehicle production as a reason for the engines being damaged. The respondents mentioned the following materials as more likely to be bitten by martens: silicon (*n* = 2) and “various organic additives” (*n* = 2). As these descriptions were largely anecdotal and not statistically relevant, the exact answers they gave can be found in the Appendix C.

In reply to questions about seasons when the interviewees serviced the most cars due to marten damage, particular seasons were mentioned repeatedly, with spring and autumn being generally mentioned the most often, irrespective of year (Table 2). Respondents in 2010 most mentioned spring as the most intense season of conflict (*n* = 13) with stone martens. In 2023, there was a significant shift in the most conflictual season, when they most commonly mentioned autumn (*n* = 11) (Table 2) (*χ* ^2^ = 9.78, df = 3, *p* = 0.02). The effect size was high (*V* = 0.46, df = 3), indicating a substantial difference. No explanations or additional comments were provided for the high occurrence of conflicts in spring during the 2010 interviews. In 2023, the reasons stated by the car mechanics behind this observation were centred around the thermal needs of the animals. “The conflicts increase during the colder periods: November, December, January, they [author's note: martens] are drawn to the still warm vehicle engines after the vehicle is parked. They use the vehicles as shelter,” one car mechanic expressed.

**TABLE 2:** Frequency of respondents (*n* = 30 in each year) reporting the highest seasonal occurrence of conflicts with stone martens in Krakow in the years 2010 and 2023.

Year	Season				Total
	Spring	Summer	Autumn	Winter	
2010	13	0	4	6	23
2023	3	2	11	5	21
Total	16	2	15	11	—

### 3.3 Types of Car Damage and Their Cost Estimates

When asked about the parts of the cars which were damaged most often, the respondents consistently mentioned similar car parts both in 2010 and 2023 (Table 3). However, there was a significant difference in the number of cases of damage to car parts (*χ* ^2^ = 9.58, df = 5, *p* = 0.002) between the years (Table 3). The effect size was high (*V* = 0.34, df = 5), indicating a substantial difference.

**TABLE 3:** Car parts reported as damaged by stone martens in the years 2010 and 2023 in Krakow.

Damaged car parts	Year	
	2010	2023
Soundproofing elements	2	15
Electrical wires	5	14
Ignition wires	5	3
Radiator tubes	2	4
Other rubber elements	7	15
Sensors	7	6
Total	28	57

In 2023, when asked to comment on the extent and costs of marten damage they encountered, the respondents gave a wide range of estimates. Most damage encountered by the respondents was minor and inexpensive to repair (such as slight wire damage), which does not immobilise the car and can be replaced for 15–20 Zloty (Polish legal tender, hereafter: PLN). The damage caused by martens was seen as more impactful in 2023 than in 2010. In 2023, respondents assessed the average cost of damages to be between approximately 400–1000 PLN, which corresponds to as much as 25% of the median net salary (3923 PLN) in Poland in 2023 (Eurostat 2026). However, more serious damages were also mentioned. Marten chewing on a single, specific wire might short‐circuit or disconnect the car's internal computer from the car's vital components and result in it no longer being able to start. One respondent said that in newer vehicles, made with more sophisticated technology, any damage to wiring that does happen has a high potential of causing very costly and serious consequences for the vehicle. “And because everything is so interconnected and fused these days, we had to pull out the entire engine, get to the computer, replace most of what was around it, really generating a lot of costs. It was many thousands PLN”‐ he recalled. These types of damages were cited as always being more costly‐ usually in the range of 2000–5000 PLN, which corresponds to as much as 127% of the median net salary in 2023. Another respondent described a situation where a marten chewed through a cable supplying electricity to the automated gearbox of a car, causing the need to replace the entire gearbox and the connected parts of the engine. The cost of this repair came to 20,000 PLN.

### 3.4 The Reported Availability, Use and Perceived Effectiveness of Mitigation Methods

In 2010, when asked if mechanics noticed any protective measures installed in the cars to counteract martens, the respondents mentioned encountering ultrasound emitters, liquid, as well as aerosol chemical repellents. Clients also used natural repellents such as animal and human hair collected from dog groomers and hairdressers, in the hope that their smell would repel the martens. None of the deterrents were pointed to as particularly popular or effective. While marten deterrents were present in the vehicles, they were mostly bought from third‐party retailers or were homemade. No deterrents were offered by the vehicle mechanics, and none were mentioned as officially sold by the vehicle manufacturers as additional features for anyone purchasing a car. In 2023, (*n* = 23) respondents reported seeing repellents used in vehicles. Out of these, a few (*n* = 6) claimed that deterrents were used very commonly and that they noticed an increase in their frequency over the years. The deterrents mentioned most often were ultrasound emitters (*n* = 17), liquid and aerosol (olfactory, chemical) repellents (*n* = 10). These were considered more standardised and reliable than homemade solutions such as dog hair (*n* = 9) and toilet blocks (*n* = 6). Two respondents also mentioned seeing homemade electric shock devices (*n* = 2). The ultrasound emitters were significantly more popular than the other countermeasures (*χ* ^2^ = 13.96, df = 4, *p* = 0.00744). The effect size was high (*V* = 0.28, df = 4), indicating a substantial difference.

When further commenting on the countermeasures, a few (*n* = 3) respondents expressed an opinion that the currently available countermeasures are effective in protecting cars from being damaged by martens, while some (*n* = 5) respondents expressed doubts about the effectiveness of these methods. Code cooccurrence analysis performed in MAXQDA showed that olfactory repellents and homemade repellents had lower reliability compared to all other types of repellents. One interviewee commented: “There are many different repellents, which might help, but it's disputable if they are effective. The best among them are the sound emitters. Olfactory repellents – among them toilet blocks installed by the drivers themselves ‐ are not very effective, because the animals get used to the smells and ignore them.” Another downside of some olfactory repellents is the way of their application. “The aerosol ones are sticky and make accessing and repairing the engine harder. It's disgusting. This [the aerosols] can even be dangerous to the car. It might make the engine heat up too much or possibly grind up the engine parts”‐ one respondent commented. Notably, another respondent remarked that, in their view, none of the countermeasures currently in use are effective at protecting cars from martens.

According to the interviewees, vehicle owners react to the damage done by martens in several ways, depending on the severity of the damage and its financial consequences. Since the damage done most often is not very severe or expensive to fix. Additionally, some vehicle owners choose not to report the damage. Two main reasons given were: the vehicle's old age, which makes the driver less likely to repair it, as they are often close to replacing them anyway, and the fact that each time an owner reports damage to a car, their insurance payments increase. This indicates that only the most debilitating damage is reported and fixed, which might influence the image and views of the conflict with stone martens. In 2010, the respondents (*n* = 18) who commented on the repellents, when asked “Does your company and/or the car's manufacturer offer any preventative measures to protect the cars from martens?” uniformly stated that there were no official repellents offered by vehicle manufacturers, whereas the rest (*n* = 12) did not offer any comments on their availability from manufacturers. All repellents used in the cars were homemade or came from third‐party companies and could be installed by the car repair services at the customer's request. The respondents in 2023 observed a general rise in client awareness of interest in marten repellents, and that clients actively search for repellents after an experience of marten car damage (*n* = 14); however, a minority (*n* = 4) claimed that the clients most often start looking to buy repellents before their car is damaged, “likely based on what they've heard from others.”

Alongside the perceived increase in awareness of the issue and growing interest in repellents, a small number of respondents (*n* = 2) also noted that vehicle manufacturers have begun offering brand‐authorised products as optional accessories through licensed car dealerships. These include the most popular types of repellents previously reported as bought almost exclusively from third‐party retailers, such as ultrasound emitters (Appendix D).

## 4 Discussion

In this study, we aimed to identify the car damage conflict with stone martens as reported by car mechanics in Krakow, the second‐largest city in Poland, in 2010 and again in 2023. We discovered that the incidence of stone marten conflicts reported by Krakow's car mechanics in 2010 has declined in 2023, despite the city's ongoing urban expansion and increase in vehicle ownership. The personal perspectives of respondents regarding the frequency of these conflicts were divided, with both favourable and unfavourable viewpoints being expressed. Furthermore, the results indicate a shift in the seasonal pattern of the conflict. While the respondents generally noticed an increase in use, availability, and sophistication of marten repellents, there was a lack of consensus about their effectiveness.

### 4.1 Perception of the Scale of the Conflict With Stone Martens

Interestingly, the number of reported conflicts significantly decreased over the decade, when we expected it to keep up with the increase in the number of cars registered in the city. An explanation for this decrease can be attributed to the fact that stone martens are territorial, and their social structures are inflexible and do not change significantly over time (Herr et al. 2009b). Moreover, the estimated stone marten population size in Krakow did not noticeably change between 2010 and 2023 (Office of Forest Management and Forest Geodesy 2016, Office of Forest Management and Forest Geodesy 2023).

Other studies conducted in Krakow within a similar timeframe (Basak et al. 2022) indicated an increase in the number of all conflicts with martens reported by the city residents, including car damage. This is in contrast with the relative decrease in conflicts reported by car mechanics in this study. It might be explained by the specific target group used by our study, focusing only on car damage, where, in fact, martens are known to cause other conflicts in urban settings, such as property intrusion and arousing fear or anxiety in the public (Basak et al. 2022). Furthermore, regarding specific stakeholders involved in studies of HWC, it was proposed by Liordos et al. (2016) that attitudes among professionally involved stakeholders are often different to those displayed by the general population. This is potentially due to the difference in the degree of exposure to the conflict and its management (Liordos et al. 2016).

Despite the reported decline in conflict frequency, some respondents indicated that the issue remained widespread and was increasingly affecting car owners. These subjective opinions can be explained by the fact that negative experiences shape human memory and thus long‐term views in a stronger way than positive ones (Barlow et al. 2012; Conner et al. 2012). In the case of car mechanics who deal with the results of this conflict often, it is possible that repeated exposure to negative experiences with stone martens caused them to subjectively feel that the conflict is more prevalent and important than it would appear, based on the numbers reported in the study. However, Perry et al. (2022) found that the influence of negative exposure might not be as strong and uniform as expected. We found that 70% of the respondents claimed to have seen general signs of marten presence in the cars, which might constitute a source of bias in their estimates of severity of the conflict among those who viewed it as very serious. Such vicarious interactions with stone martens do not necessarily imply the existence of conflict but requires socioeconomic context (Young et al. 2010). High estimated cost of repair and the repetitive nature of the problem may result in negative interpretations of seeing traces of marten presence in the vehicles serviced. Additionally, the respondents in this study who did not consider the conflict to be a big issue used dismissive language which minimises the severity of the conflict, such as claiming that the damage done to car engines is incidental or mostly aesthetic. This might suggest that the stone martens are a recurring nuisance but not strictly a conflict species in central‐eastern Europe, which is supported by other research done on human‐wildlife conflicts in urban ecosystems (Moesch et al. 2024). One of the interviewees mentioned that his clients seem to believe that the conflict is widespread, despite not encountering it often himself. While it is only an one comment and cannot be used to construct a narrative encompassing all opinions and attitudes of both car mechanics and drivers, it does suggest a high impact of perceived risk and nuisance on overall disposition of the drivers (Slovic 2015).

### 4.2 Views on Factors Potentially Causing or Influencing the Occurrence of Car Damage

The results of this research indicate that there was a change in the perceived seasonality of the conflict during the decade separating the two rounds of interviews. In 2010, stone martens caused the most conflict in spring, while in 2023, they caused the most conflict in autumn. These results were also consistent with Herr et al. (2009a), who found that autumn, spring, and summer were the times when stone martens caused the most conflict during a year‐long study. Assuming the intensity of the conflict is influenced by the animals' activity patterns, both periods of increased frequency of conflicts can be explained by the ecology of the species and could reasonably co‐occur within the same year. Spring is the time when stone martens, especially females, are increasingly territorial and likely more mobile and active (Herr and Roper 2022). In autumn, young stone martens enter adulthood and disperse, occupying new territories, which often partially overlap with the territories of other martens of the same sex or overlap completely with the territories of martens of the opposite sex (Okarma and Tomek 2008). The seasonal variation in reported conflict intensity may also suggest that the issue is highly dynamic and continuously changing, with the potential for stone marten conflict appearing to be independent of the time of year.

Territoriality and territory‐marking behaviours are potential factors causing the conflict with stone martens (Herr et al. 2009a). According to some of the respondents, anywhere between 50% and 70% of cars serviced in Krakow have traces of marten activity in them, which possibly indicates that they are indeed used by martens for marking their territories, either purposefully or incidentally when searching for sheltered feeding spaces. The daily use of marked cars by humans for travel between work and home might bring them into contact with other stone martens, who could potentially react with aggression, stress, and trigger territorial behaviour (Herr et al. 2009a).

### 4.3 Types of Car Damages and Their Cost Estimates

Results show a change in the frequencies of car elements reported as being damaged most often, with a large increase in the number of mentions of soundproofing or muffling elements. The results indicate a shift in the frequency of car components reported as being most frequently damaged, with a notable increase in mentions of soundproofing or muffling elements. The increase in the number of damages to engine muffling elements, such as thick fabric screens and rubber mats, can be explained by the fact that car manufacturers installed more of them in recent models as compared to older car models produced before 2010. In 2023, the interviewees also started pointing to the chemical makeup and possibly the smell of the car parts for the first time as a possible factor in the targeting of particular car models or car manufacturer brands. We found no studies which could offer any proof or explanation for this supposed phenomenon or other examples of such statements; therefore, these claims should be further verified with additional surveys or experimental tests. Nevertheless, should the claims prove to be rooted in reality, they open yet another avenue of potential research into the causes of and mitigation strategies in the conflict with stone martens. In 2023, the costs mentioned by the car mechanics are a relatively high percentage of the median salary in the country, providing further reasons for why the conflict's image is that of a persistent one.

### 4.4 The Reported Availability, Use and Perceived Effectiveness of Mitigation Methods

Interviewees highlighted the ongoing use and search for marten repellents by their customers. The respondents believe that these measures are only partially effective in conflict mitigation, and they also expressed concerns about their actual usefulness and potentially detrimental effect on the car engines themselves. These concerns seem to be connected primarily with negative experiences with home‐made and olfactory (chemical) repellents and are the foundation of most negative opinions about all repellents, regardless of type, which is again consistent with the idea of negative experiences that is, with sticky aerosol‐applied repellents, having a stronger influence on opinions and memory than neutral or positive ones, that is, with other, potentially better countermeasures (Barlow et al. 2012; Conner et al. 2012).

A new development in 2023 was the inclusion of officially licensed marten repellents by the car manufacturers, showing that they view the issue as significant. It is likely that the observed decrease in existing marten‐caused car damage incidents is related to this development and increase in repellent sales and sophistication. Car owners are more aware of and sensitive to the risk of damage and often seek countermeasures after experiencing it, indicating that they consider it a growing threat, possibly more so than the car mechanics.

### 4.5 The Conflict and Its Mitigation as Basis for Understanding Human Relationships With Stone Martens

This is the first study to examine human–marten conflict in Poland. The topic has received very limited attention in both public discourse and scientific research, making this work pioneering in the national context. By identifying and characterising the nature of these conflicts, the study provides a foundation for future research and may help inform the development of evidence‐based management and urban planning strategies, similar to those increasingly considered for urban wild boar management. Tolerance toward stone martens may be increased by reducing opportunities for conflict and encouraging them to use areas where their presence is more acceptable for humans. People tend to tolerate wildlife more when it occurs in locations they perceive as appropriate, whereas animals found outside of their perceived place—and especially close to human property—are often viewed less favourably (Sweet et al. 2024). Providing better living spaces for the martens and additional denning and resting areas in places perceived as acceptable for them to inhabit, such as urban green areas, might reduce their interactions with parked cars and territorial behaviours (O'donnell and de Nicola 2006). Other marten species were also shown to use artificial shelter more readily than other animals (Delheimer et al. 2018), making them ideal for marten conservation and management, with reduced risk of affecting non‐target species. Measures such as installing wooden boxes as shelter were proven to enrich the environment for pine martens and reduce conflicts in the United Kingdom (Croose et al. 2016), which could prove useful in urban environments, in places such as parks and internal green spaces to not only keep the animals in human‐accepted spaces but also provide them with alternatives to interacting with cars and damaging them when exhibiting territorial behaviours. Combining the knowledge of public attitudes with an understanding of stone marten spatial ecology and use of urban areas could help identify areas best suited for informed urban planning measures that reduce stone marten interactions with humans and human property.

### 4.6 Recommendations for Stone Marten Management and Decision Makers

Urban residents are able to mitigate wildlife conflicts by taking both individual and coordinated action. However, adoption of these measures is more likely when residents perceive them as necessary and effective, particularly if they have previous conflict experience or concerns about zoonotic diseases (Puri et al. 2024). Education campaigns on available mitigation tools and actions which reduce stone marten attraction to vehicles and improve greenspace connectivity should therefore play an important role in promoting coexistence by increasing awareness of practical and effective conflict‐prevention strategies and their application for avoiding the conflict.

## 5 Limitations and Recommendations for Future Research

The wide range of responses and uncertainty expressed among car mechanics regarding both the frequency of the conflict with stone martens and existing mitigation measures illustrates an important knowledge gap in our understanding of this issue. This study should be treated as a starting point for further, more in‐depth research on human–stone marten relations. Our investigation was exploratory and focused on one group of stakeholders situated at the interface of a potential conflict. While we did not examine the broader social dimensions of this issue, such as values, attitudes, tolerance, or emotional responses, our analysis of reported damages and mechanics' opinions indicates that the phenomenon is shaped by beliefs, perceptions, and other social variables. A comprehensive understanding of the situation therefore requires additional interdisciplinary research—both social studies and ecological assessments of stone marten populations in urban environments. Future studies should include car drivers as a key stakeholder group and investigate the reasons for reporting or not reporting damages, as well as attitudes and emotions associated with these events. Using questionnaires in addition to interviews can be a good way of illustrating the views and needs of stakeholders on a large scale while also providing researchers with a wide range of citizens' and organisations' views and ideas regarding HWC (Marshall et al. 2007).

The emerging questions regarding the role of the chemical make‐up of materials used in car engine manufacturing, as well as the effectiveness and safety of using currently available marten repellents, open another avenue of potential future research. Comprehensive studies involving car manufacturers, mechanics and owners can make our understanding of HWCs in a more complete way (Pătru‐Stupariu et al. 2020), combining efforts to address this HWC by finding effective mitigation measures. Local stakeholder experience can be a useful source of knowledge used to change conservation policy, making it more participatory and socially involved (Thaman et al. 2013). In the specific case of the relations between humans and stone martens, the use of knowledge of car mechanics and other stakeholders could be a starting point in any future research which aims to provide ways of reducing the nuisance status of the species and facilitating better human‐wildlife coexistence.

## 6 Conclusion

This paper is, to our knowledge, the first one to set out to identify the stone marten conflict in an urban setting by examining a decade of changes in the conflict through the experiences and views of car mechanics. In the eyes of some car mechanics, the conflict is persistent, and their views are polarised both concerning the frequency and effectiveness of the existing mitigation methodologies. Understanding the perspectives of professional stakeholders can aid in improving the existing countermeasures and strategies for stone marten conflict management and direct future studies. Although the conducted interviews cannot represent all aspects of the conflict or the experiences of all the car mechanics in Krakow, they provide a rich source of local knowledge which can be used in the management of the conflict and HWC, as well as give a baseline for comparison in future studies.

## Author Contributions

**Michał Strączyński:** conceptualization (equal), data curation (equal), formal analysis (equal), investigation (equal), methodology (equal), writing – original draft (equal), writing – review and editing (equal). **Sayantani M. Basak:** methodology (equal), supervision (equal), writing – review and editing (equal). **Paweł Kwapisz:** data curation (equal), investigation (equal), writing – review and editing (equal). **Joanna Tusznio:** software (equal), validation (equal), writing – review and editing (equal). **Jesse S. Lewis:** writing – review and editing (equal). **Izabela A. Wierzbowska:** conceptualization (equal), supervision (equal), writing – review and editing (equal).

## Funding

The authors have nothing to report.

## Conflicts of Interest

The authors declare no conflicts of interest.

## Supporting information


**Appendix A:** Standard questions asked the interviewees and their English translations.
**Appendix B:** Localisations of car mechanic services surveyed in 2010 and 2023 and the frequencies of conflicts they reported.
**Appendix C:** Answers given to questions related to the interviewees' views on factors potentially causing or influencing the car damage.
**Appendix D:** Repellents officially authorised and sold by two car manufacturers, advertised in two separate car mechanic services (D1) and (D2).

## Data Availability

All the required data are uploaded as Supporting Information S1.
