# Supplementary material for: Urban Stone Marten (Martes foina)–Human Conflicts: A Longitudinal Survey‐Based Approach to a Persistent Challenge
Source: Ecol Evol. 2026 Sep 4;16(9):e74294. doi: 10.1002/ece3.74294 (PMC13542989; doi:10.1002/ece3.74294)
Supplement: Supplementary file 1 [file ECE3-16-e74294-s001.docx]

Appendix A. Standard questions asked the interviewees and their English translations.

| Original question in Polish | English translation |
| --- | --- |
| 1. Ile samochodów serwisowaliście państwo w 2022 roku? Czy macie dane zbiorcze z wcześniejszych lat, np. ostatniej dekady?  1a. Jaki procent samochodów został uszkodzony przez kuny? | 1. How many cases were brought into this mechanic workshop this year, how many do you usually per year?  1a. Out of these, how many were damaged by martens? |
| 2. Kiedy najczęściej dochodzi do uszkodzenia samochodu przez kunę? W jakich miesiącach serwisowano najwięcej samochodów? | 2. When did most of the marten damage occur? In which months was the majority of the cars serviced? |
| 3. Które modele samochodów kuny uszkadzały najczęściej? Czy zaobserwowaliście państwo zależność od wieku? | 3. What car models were brought in because of martens the most/least often? Did you notice if there was any influence of the car’s age? |
| 5. Które części samochodu/silnika były uszkadzane najczęściej?  5a. Jakie były koszty zniszczeń i naprawy? | 5. What parts of the car were damaged the most often?  5a. What are the costs of the damages? |
| 6. Czy ma Pan/Pani dodatkowe obserwacje dotyczące uszkodzeń dokonanych przez kuny? | 6. Do you have any additional observations regarding the damage? |
| 7. Gdzie parkowane były uszkodzone samochody (Okolice terenów zielonych, centrum, otwarte parkingi czy garaże)? | 7. Where were the damaged cars parked most often? |
| 8. Czy w samochodach zainstalowane były środki odstraszające kuny, takie jak emitery dźwięków, produkty Kunagone itp? | 8. Were there any protective measures installed in the cars? Such as sound emitters, Kunagone etc.? |
| 9. Czy państwa serwis i/lub producent samochodu oferuje jakiekolwiek środki odstraszające kuny? Jeśli tak to jakie?  9a. Jeśli oferujecie państwo takie środki, jak wygląda popyt na nie wśród klientów przed i po uszkodzeniu auta przez kuny? | 9. Does your business and/or the car’s manufacturer offer any repellents to protect the cars from martens?  9a. If you do sell the repellents, did you notice if the demand for them increases after a person’s car was damaged or do they search for them pre-emptively? |

Appendix B Localisations of car mechanic services surveyed in 2010 and 2023 and the frequencies of conflicts they reported

A= Respondents in 2010; B= Respondents in 2023

| **ID** | **Address** | **ID** | **Address** |
| --- | --- | --- | --- |
| A1 | Aleja Pokoju | B1 | Aleja Pokoju |
| A2 | Armii Krajowej | B2 | Armii Krajowej |
| A3 | Opolska | B3 | Opolska |
| A4 | Ofiar Dąbia | B4 | Ofiar Dąbia |
| A5 | Profesora Adama Rożańskiego | B5 | Profesora Adama Rożańskiego |
| A6 | Profesora Adama Rożańskiego | B6 | Profesora Adama Rożańskiego |
| A7 | Josepha Conrada | B7 | Josepha Conrada |
| A8 | Łokietka | B8 | Łokietka |
| A9 | Góra Libertowska | B9 | Góra Libertowska |
| A10 | Góra Libertowska | B10 | Góra Libertowska |
| A11 | Zakopiańska | B11 | Zakopiańska |
| A12 | Aleja Pokoju | B12 | Aleja Pokoju |
| A13 | Zakopiańska | B13 | Zakopiańska |
| A14 | Góra Libertowska | B14 | Skotnicka |
| A15 | Zakopiańska | B15 | Tyniecka |
| A16 | Al.29 Listopada | B16 | Zakopiańska |
| A17 | Al. 29 Listopada | B17 | Stanisława Klimeckiego |
| A18 | Jasnogórska | B18 | Świetlista |
| A19 | Zakopiańska | B19 | Rostworowskiego |
| A20 | Josepha Conrada | B20 | Bolesława Leśmiana |
| A21 | Jasnogórska | B21 | Księcia Józefa |
| A22 | Jasnogórska | B22 | Pylna |
| A23 | Jasnogórska | B23 | Rostworowskiego |
| A24 | Jasnogórska | B24 | Królowej Jadwigi |
| A25 | Powstańców Śląskich | B25 | Centralna |
| A26 | Wielicka | B26 | Rydlówka |
| A27 | Balicka | B27 | Twardowskiego |
| A28 | Ofiar Dąbia | B28 | Siwka |
| A29 | Zawiła | B29 | Na Załęczu |
| A30 | Radzikowskiego | B30 | Radzikowskiego |

Appendix C Answers given to questions related to the interviewees’ views on factors potentially causing or influencing the car damage

| Question: | Answers provided: | |
| --- | --- | --- |
|  | 2010 | 2023 |
| “Which car models were brought in because of martens the most/least often?” “Do you see a pattern of older or newer cars being attacked more often?” | Neither model or the age of the car influenced the conflict | “The older vehicles have much fewer soundproofing elements in the engine compartments than the newer cars, thus the martens overwhelmingly target the various electrical wires.” Additionally, “New vehicles, regardless of model were targeted more often in general because of the intense smell of new plastic and rubber parts, which attracts the animals”. Most interviewees (n= 15) however claimed that the age of the vehicle does not influence the conflict in any way and suggested other factors.  “Modern cars might be harder to get to and damaged. These days, everything (the engine) is just so enclosed and separated from the outside that it must make it harder for the marten to get in. Electric cars, oh- they are very safe, I think. There are barely any openings in the ones we have seen over here for instance. And, obviously, if the marten cannot get to the engine, it is unlikely it will damage it.” |
| Do you know where were the damaged cars were parked? | “It is one of the most important factors influencing the conflict” | “The place a vehicle is parked is not significant to the risk, especially since the cars leave the garages during the day when the owners drive to work and other places and are often left in the open.” |
| Do you have any additional observations regarding the damage? | The interview participants in 2010 did not describe additional car-related factors. | -On the topic of the general role of materials used in car manufacturing:  “Certain vehicle manufacturers might use specific admixtures in their engine elements which attract the martens”  -On the topic of the use of silicon:  “[…] the cables covered in it seem to be targeted more often, simply because the martens like to bite into it. It’s the taste […]”.  -On the topic of “Various organic additives”:  “It is said that some car manufacturers use plant-sourced substances in their composite materials, possibly as a way of recycling the organic matter or making the parts cheaper and less damaging for the environment, usually in the form some sort of soy product. Because the parts are made from plant-sourced materials, they might give off the smell of food that the martens pick up on and start chewing.” |

Appendix D Repellents officially authorised and sold by two car manufacturers, advertised in two separate car mechanic services (D1) and (D2)

D1

D2
